# Comprehensive atrial fibrillation burden and symptom reduction post-ablation: insights from DECAAF II

**DOI:** 10.1093/europace/euae104

**Published:** 2024-04-22

**Authors:** Charbel Noujaim, Ala Assaf, Chanho Lim, Han Feng, Hadi Younes, Mario Mekhael, Nour Chouman, Ghaith Shamaileh, Abdel Hadi El Hajjar, Tarek Ayoub, Nino Isakadze, Mihail G Chelu, Nassir Marrouche, Eoin Donnellan

**Affiliations:** Tulane Research Innovation for Arrhythmia Discovery, 1430 Tulane Avenue, New Orleans, LA, USA; Tulane Research Innovation for Arrhythmia Discovery, 1430 Tulane Avenue, New Orleans, LA, USA; Tulane Research Innovation for Arrhythmia Discovery, 1430 Tulane Avenue, New Orleans, LA, USA; Tulane Research Innovation for Arrhythmia Discovery, 1430 Tulane Avenue, New Orleans, LA, USA; Tulane Research Innovation for Arrhythmia Discovery, 1430 Tulane Avenue, New Orleans, LA, USA; Tulane Research Innovation for Arrhythmia Discovery, 1430 Tulane Avenue, New Orleans, LA, USA; Tulane Research Innovation for Arrhythmia Discovery, 1430 Tulane Avenue, New Orleans, LA, USA; Tulane Research Innovation for Arrhythmia Discovery, 1430 Tulane Avenue, New Orleans, LA, USA; Tulane Research Innovation for Arrhythmia Discovery, 1430 Tulane Avenue, New Orleans, LA, USA; Tulane Research Innovation for Arrhythmia Discovery, 1430 Tulane Avenue, New Orleans, LA, USA; Department of Cardiovascular Medicine, Johns Hopkins University School of Medicine, Baltimore, MD, USA; Department of Internal Medicine, Baylor College of Medicine, Houston, TX, USA; Division of Cardiology, Baylor College of Medicine, Houston, TX, USA; Baylor St Luke’s Medical Center, Houston, TX, USA; Texas Heart Institute, Houston, TX, USA; Tulane Research Innovation for Arrhythmia Discovery, 1430 Tulane Avenue, New Orleans, LA, USA; Tulane Research Innovation for Arrhythmia Discovery, 1430 Tulane Avenue, New Orleans, LA, USA

**Keywords:** Atrial fibrillation, AF burden, Smartphone ECG, Catheter ablation, Fibrosis, AF symptoms

## Abstract

**Aims:**

Traditional atrial fibrillation (AF) recurrence after catheter ablation is reported as a binary outcome. However, a paradigm shift towards a more granular definition, considering arrhythmic or symptomatic burden, is emerging. We hypothesize that ablation reduces AF burden independently of conventional recurrence status in patients with persistent AF, correlating with symptom burden reduction.

**Methods and results:**

Ninety-eight patients with persistent AF from the DECAAF II trial with pre-ablation follow-up were included. Patients recorded daily single-lead electrocardiogram (ECG) strips, defining AF burden as the proportion of AF days among total submitted ECG days. The primary outcome was atrial arrhythmia recurrence. The AF severity scale was administered pre-ablation and at 12 months post-ablation. At follow-up, 69 patients had atrial arrhythmia recurrence and 29 remained in sinus rhythm. These patients were categorized into a recurrence (*n* = 69) and a no-recurrence group (*n* = 29). Both groups had similar baseline characteristics, but recurrence patients were older (*P* = 0.005), had a higher prevalence of hyperlipidaemia (*P* = 0.007), and had a larger left atrial (LA) volume (*P* = 0.01). There was a reduction in AF burden in the recurrence group when compared with their pre-ablation burden (65 vs. 15%, *P* < 0.0001). Utah Stage 4 fibrosis and diabetes predicted less improvement in AF burden. The symptom severity score at 12 months post-ablation was significantly reduced compared with the pre-ablation score in the recurrence group, and there was a significant correlation between the reduction in symptom severity score and the reduction in AF burden (*R* = 0.39, *P* = 0.001).

**Conclusion:**

Catheter ablation reduces AF burden, irrespective of arrhythmia recurrence post-procedure. There is a strong correlation between AF burden reduction and symptom improvement post-ablation. Notably, elevated LA fibrosis impedes AF burden decrease following catheter ablation.

## Graphical Abstract

**Graphical Abstract:**
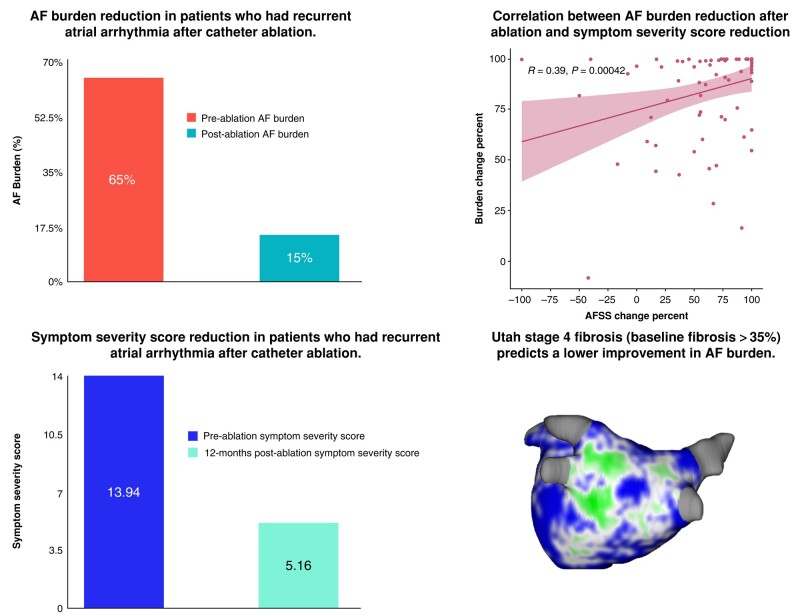

**What’s new?**

The study introduces a nuanced perspective on the outcomes of catheter ablation, emphasizing not just the binary metric of arrhythmia recurrence but also the more granular aspects such as arrhythmic burden and symptom severity, as well as an innovative and effective monitoring strategy, that is, smartphone-based single-lead electrocardiogram technology. This approach aligns with the evolving paradigm in electrophysiology that seeks to understand atrial fibrillation (AF) management outcomes beyond mere recurrence rates, highlighting a significant reduction in AF burden and symptom severity post-procedure. Clinicians can appreciate that lower pre-ablation left atrial fibrosis levels may predict better ablation outcomes.

## Introduction

The assessment of catheter ablation outcome is traditionally based on a binary definition of atrial arrhythmia recurrence, which determines the presence or absence of atrial fibrillation (AF) episodes following a 90-day blanking period. However, recent studies have recommended a shift towards a more inclusive and meaningful outcome, known as AF burden.^1^ Various methods exist for determining AF burden, one of which involves calculating the proportion of time a patient experiences AF during a specified duration. This approach facilitates the generation of a continuous, or quasi-continuous, representation of the patient’s dynamic arrhythmic profile.

The evaluation of AF burden before and after catheter ablation has the potential to provide a more nuanced and detailed measurement of treatment efficacy than the binary recurrence definition. The latter only detects the presence or absence of AF episodes, while the former considers the amount of AF, offering a more objective measurement of improvement. Moreover, even in cases of AF recurrence, catheter ablation has been linked to an improvement in the quality of life and symptom burden.^2^

This study aims to propose a non-invasive method of evaluating AF burden using a smartphone single-lead electrocardiogram (ECG) device (SMURDEN). We hypothesize that catheter ablation decreases SMURDEN independently of conventional recurrence status in the persistent AF population, and a decrease in SMURDEN correlates with a reduction in symptom burden.

## Methods

### Study population

Details of the DECAAF II trial have been published.^3^ In short, the DECAAF II trial was a large investigator-initiated, industry-sponsored, prospective, multicentre (44 sites, 3 continents), randomized controlled clinical trial in which 843 patients with persistent AF were randomized into two treatment arms comparing magnetic resonance imaging (MRI)-guided fibrosis ablation + pulmonary vein isolation (PVI) vs. PVI alone. To be enrolled in the trial, patients had to have persistent AF (defined as 7 days or more of AF as evidenced by either a rhythm strip or documentation on a chart review) and must have been undergoing their first AF ablation. The main exclusion criteria were contraindication to gadolinium and/or MRI and previous AF ablation or valvular cardiac surgery. This study was approved by the Tulane University Biomedical IRB.

### Electrocardiogram acquisition

All patients received a handheld smartphone ECG device (ECG Check, Cardiac Designs^4^) and were required to record ECG strips daily and in case of any relevant symptoms during the study follow-up period. Ambulatory monitoring and 12-lead ECG data performed as part of clinical care were also reported as components of the primary outcome of the original trial, but are not part of this particular analysis. Electrocardiogram strips from the handheld device were transmitted automatically to the ECG core laboratory for analysis by trained experts masked from treatment assignment. A number of patients started submitting ECG strips for analysis from the initial screening visit. Electrocardiogram strips were intended for patient follow-up after the procedure, and pre-ablation rhythm monitoring was not required. For this analysis, which focuses on the difference between pre- and post-ablation arrhythmic burden, we included only those patients who recorded at least 10 ECG strips in the pre-ablation period.

### Catheter ablation procedure

#### Fibrosis-guided ablation

For patients randomized to the fibrosis-guided ablation group, processed delayed-enhancement MRIs were merged with the 3D mapping system at each study site to be used during the procedure. All patients underwent PVI. After PV entrance block had been confirmed, fibrosis-guided ablation was pursued. The operator either encircled or covered with ablation lesions all fibrotic areas observed on delayed-enhancement MRI. Details regarding the ablation protocol for both treatment groups are included in the main manuscript.

#### Pulmonary vein isolation

All PVs were electrically isolated, as described by the Heart Rhythm Society Consensus Statement.^5^ If normal sinus rhythm could not be restored, despite cardioversion at the end of the PVI portion of the procedure in patients randomized to this group, the operator had the option to pursue other measures to eliminate recurrent arrhythmias if needed.

### Imaging

Patients underwent a delayed-enhancement MRI within 30 days prior to the ablation procedure using the Merisight delayed-enhancement MRI protocol (MARREK Inc.). The purpose of the baseline MRI was to quantify left atrial (LA) fibrosis in all patients. Patients’ randomized treatment group was masked from reviewers who assessed MRI quality. MARREK Inc. assisted with image segmentation, processing, and quantification of LA fibrosis. Following ablation, delayed-enhancement MRIs were obtained at 90–180 days to quantify ablation-related scar formation.

### Primary outcome

The primary endpoint of the study was the first confirmed recurrence of atrial arrhythmia (including AF, atrial flutter, or atrial tachycardia) lasting for at least 30 s after the 90-day blanking period, demonstrated by single-lead smartphone ECG device tracing, one positive reading on a clinical 12-lead ECG tracing, ambulatory monitor, or if the patient underwent repeat ablation, over a follow-up period of 12–18 months. The daily smartphone ECGs were intended as the primary method for assessing atrial arrhythmia recurrence, but clinical and ambulatory ECGs served as back-up methods for detecting recurrence in patients who failed to reliably transmit smartphone ECG readings. A core laboratory at the University of Washington adjudicated the ECG findings.

### Smartphone atrial fibrillation burden (SMURDEN)

Atrial fibrillation burden was defined as the proportion of days on which the submitted ECG strips showed evidence of AF, out of the total number of days on which ECG strips were submitted for each patient during a specified period. The AF burden was calculated from the time of initial screening until the day of ablation, and from the end of the blanking period until the end of follow-up. If multiple ECG strips were provided on the same day, and all showed sinus rhythm, that day was considered a sinus rhythm day. However, if at least one ECG strip showed evidence of AF on that day, the day was classified as an AF day.

### Atrial fibrillation severity scale

The AF severity scale (AFSS) is a self-administered questionnaire comprising 19 items, developed to measure both subjective and objective ratings of symptoms related to AF, healthcare utilization, and the overall burden of AF, including the frequency, duration, and severity of episodes. For the purposes of this analysis, we focused on Part C of the questionnaire, which specifically assesses the frequency of AF-related symptoms such as palpitation, shortness of breath at rest and during physical activity, exercise intolerance, fatigue at rest, lightheadedness/dizziness, and chest pain/pressure. The severity of each symptom is rated on a 6-point scale, ranging from 0 to 5 points. The resulting scores range from 0 to 35 points, with higher scores indicating greater AF symptom severity. The AFSS questionnaire was administered at the screening visit and 12 months’ post-ablation.

### Statistics

The report of all continuous variables is given as the mean ± standard deviation (SD), with comparison achieved through Student’s *t*-tests and Wilcoxon tests, dependent on the outcome of the normality assumption verification via the Shapiro–Wilk test. For categorical variables, they are detailed as either percentages or frequencies, with comparisons made through *χ*^2^ tests.

An exploration of univariate analyses was undertaken to identify potential predictors of the AF burden post-ablation from a group of variables of interest. The outcomes of these analyses are presented as regression coefficients (*β*). Inclusion in a multivariate model was considered for variables with a *P*-value of <0.3, using a stepwise selection process, where the threshold for inclusion was 0.05. For categorical variables with multiple levels, we combined the non-significant levels to obtain a parsimonious model.

The non-linear relationship between the change in AF burden and the change in AFSS score among the patients who underwent ablation was evaluated using the Spearman correlation coefficient. The entire statistical analyses was carried out using R 4.2.0 (http://www.R-project.org, The R Foundation), employing a two-sided significance level of 0.05 as the standard.

## Results

### Baseline characteristics

We included 98 patients who underwent pre-ablation ECG monitoring and submitted more than 10 single-lead ECG strips for analysis during that time period. The study population had a mean age of 61.6 years (SD = 9.3), with 72.4% being males, and a mean baseline LA fibrosis of 18.89% (SD = 7.08). At the end of the follow-up period, 69 patients had atrial arrhythmia recurrence, while 29 remained in sinus rhythm. We categorized these patients into a no-recurrence group (Group 1, *n* = 29) and a recurrence group (Group 2, *n* = 69). Overall, baseline characteristics, comorbidity profile, and medication history were similar between the two groups. Nevertheless, patients in Group 2 tended to be older (63.2 vs. 57.9 years, *P* = 0.005), had a higher prevalence of hyperlipidaemia (37.7 vs. 10.3%, *P* = 0.007), and a larger LA volume (111.4 vs. 92 mL, *P* = 0.01). The mean follow-up from screening to ablation in our population was 62 days. There was a significant difference in pre-ablation follow-up times between the two groups (55.9 vs. 75.6 days, *P* = 0.01). Additionally, the number of days with ECG strip submission in this period (pre-ablation) was significantly different between the two groups (51.7 vs. 37.5, *P* = 0.02). However, the proportion of days with ECG submissions from screening to ablation was similar between the two groups (68 vs. 67%, *P* = 0.74). There was no correlation between pre-ablation ECG submission rate and pre-ablation AFSS (*r* = 0.16, *P* = 0.12) or pre-ablation AF burden (*r* = 0.03, *r* = 0.78). *Table 1* presents baseline characteristics, AFSS, and single-lead ECG data.

**Table 1:** Baseline characteristics

	No-recurrence group (*n* = 29)	Recurrence group (*n* = 69)	Total (*n* = 98)	*P*-value
Age (years)	57.9	63.2	61.6	0.005
Males (%)	23 (79.3)	48 (69.6)	71 (72.4)	0.32
Baseline fibrosis (%)	18.04	18.97	18.7	0.44
Utah stage				0.35
I	2 (6.9)	9 (13)	11 (11.2)	
II	18 (62.1)	30 (43.5)	48 (49)	
III	6 (20.7)	23 (33.3)	29 (29.6)	
IV	3 (10.3)	7 (10.1)	10 (10.2)	
Treatment received				0.06
PVI	19	31	50	
PVI + fibrosis-guided ablation	10	38	48	
Hypertension	17 (58.6)	37 (53.6)	54 (55.1)	0.64
Diabetes	3 (10.3)	8 (11.6)	11 (11.2)	0.85
CHF	7 (24.1)	13 (18.8)	20 (20.4)	0.55
Stroke	2 (6.9)	7 (10.1)	9 (9.2)	0.61
Vascular disease	2 (6.9)	5 (7.2)	7 (7.1)	0.95
Mitral regurgitation	3 (10.3)	4 (5.8)	7 (7.1)	0.42
Tobacco	15 (51.7)	28 (40.6)	43 (43.9)	0.31
CAD	0 (0)	8 (11.6)	8 (8.2)	0.06
Hyperlipidaemia	3 (10.3)	26 (37.7)	29 (29.6)	0.0068
BMI	31.07	33.06	32.5	0.21
AADs	12 (41.4)	30 (43.5)	42 (42.9)	0.84
Statins	3 (10.3)	27 (39.1)	30 (30.6)	0.0048
Failed AAD treatment	16 (55.2)	29 (42)	45 (45.9)	0.23
LA volume (mL)	92	111.4	105.6	0.01
Pre-ablation AFSS	16.52	13.94	14.7	0.11
1-Year post-ablation AFSS	3.24	5.16	4.59	0.02
AFSS decrease	13.28	8.76	10.11	0.02
Pre-ablation follow-up (days)	75.6	55.9	61.7	0.01
Pre-ablation number of days with ECG submission	51.7	37.5	41.7	0.02
Proportion of days with ECG submission over pre-ablation follow-up	0.68	0.67	0.67	0.74
Post-ablation number of days with ECG submission	163.4	281.7	246.7	0.0002

AAD, antiarrhythmic drug; AFSS, atrial fibrillation severity scale; BMI, body mass index; CAD, coronary artery disease; CHF, congestive heart failure; ECG, electrocardiogram; LA, left atrium.

### Delta atrial fibrillation burden

**Figure 1:**
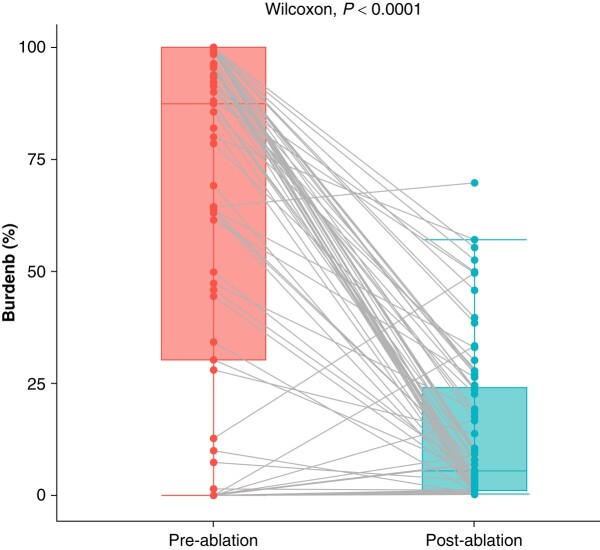
Atrial fibrillation burden reduction in patients who had recurrent atrial arrhythmia after catheter ablation. The grey lines represent individual patients.

The pre-ablation AF burden was similar in both groups (59 vs. 65%, *P* = 0.58). At the conclusion of the observation period, the no-recurrence cohort demonstrated an anticipated post-ablation AF burden of 0%, while the recurrence cohort had a burden of 15%. Of note, there was a reduction in AF burden in the recurrence group when compared with their pre-ablation burden (65 vs. 15%, *P* < 0.0001), as illustrated in *Figure 1*. We performed additional analysis to calculate the AF burden after the first non-blanking period AF event, and compared it with the overall AF burden previously reported, and we found no significant difference (15 vs. 16.4%, *P* = 0.48).

### Predictors of atrial fibrillation burden decrease

Univariate and multivariate predictors of AF burden decrease are presented in *Table 2*. Multivariate analysis showed that a higher pre-ablation AF burden is associated with a higher decrease in AF burden. Conversely, Utah Stage 4 fibrosis (baseline fibrosis > 35%) and diabetes predicted a lower improvement in AF burden.

**Table 2:** Univariate and multivariate analysis of predictors of post-ablation AF burden

	Effect	SE	*P*-value
*Univariate analysis*			
Pre-ablation AF burden	0.86	0.084	<0.0001
Treatment received (PVI only vs. PVI + MRI)	0.074	0.079	0.35
Age	0.0020	0.0040	0.62
Sex	−0.023	0.083	0.78
Baseline Utah stage			0.13
I	Reference	Reference	Reference
II	0.043	0.12	
III	0.15	0.13	
IV	−0.13	0.16	
Baseline terminal Utah Stage IV (compared with any other Utah stage)	−0.22	0.12	0.07
AAD	−0.15	0.078	0.069
ARB	−0.18	0.089	0.053
Statins	0.024	0.083	0.78
CHF	−0.046	0.098	0.64
HTN	0.060	0.079	0.45
DM	−0.19	0.11	0.11
Stroke	−0.010	0.14	0.94
Vascular disease	0.16	0.17	0.36
Tobacco	0.028	0.082	0.73
CAD	−0.20	0.11	0.082
Hyperlipidaemia	−0.026	0.083	0.76
Failed AAD	−0.16	0.077	0.039
BMI	0.0042	0.0059	0.48
LA volume	0.000011	0.0011	0.99
Pre-ablation AFSS	0.010	0.0053	0.059
Mitral valve	0.0087	0.15	0.95
*Multivariate analysis*			
Intercept	0.018	0.063	0.77
Pre-ablation AF burden	0.84	0.073	<0.0001
Baseline Utah Stage IV	−0.18	0.063	0.0057
DM	−0.19	0.059	0.0019

AAD, antiarrhythmic drug; AF, atrial fibrillation; AFSS, atrial fibrillation severity scale; ARB, angiotensin receptor blocker; BMI, body mass index; CAD, coronary artery disease; CHF, congestive heart failure; DM, diabetes mellitus; HTN, hypertension; LA, left atrium; MRI, magnetic resonance imaging; PVI, pulmonary vein isolation; SE, standard error.

### Atrial fibrillation severity scale

**Figure 2:**
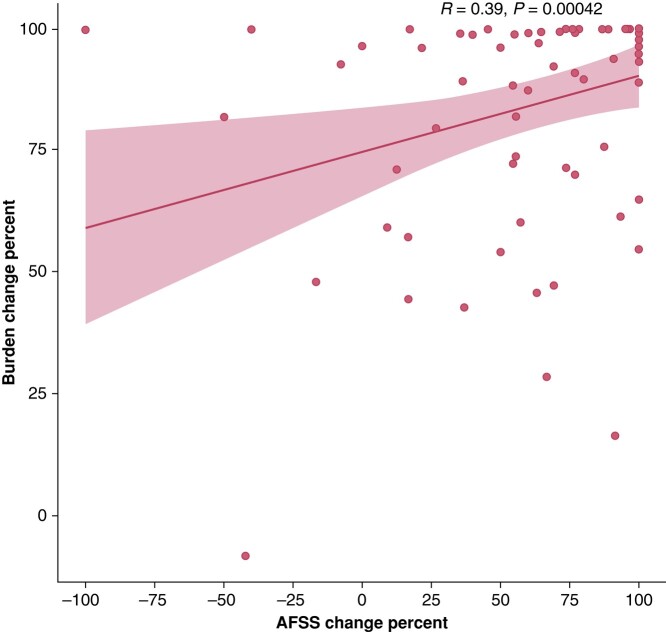
Correlation between atrial fibrillation burden variation and symptom severity variation.

The pre-ablation symptom severity score was comparable between the two groups (16.5 vs. 13.9, *P* = 0.11). However, the 12-month post-ablation symptom severity score was significantly lower in the no-recurrence group when compared with the recurrence group (3.24 vs. 5.16, *P* = 0.02). Notably, the symptom severity score at 12 months post-ablation was significantly reduced compared with the pre-ablation score in the recurrence group (13.94 vs. 5.16, *P* = 0.01). Additionally, there was a significant correlation between the decrease in symptom severity score and the decrease in AF burden after the ablation (*R* = 0.39, *P* = 0.001), as illustrated in *Figure 2*.

We developed two multivariable models to predict post-ablation AFSS. Model 1 uses AF recurrence status, time to recurrence, and post-ablation AF burden. Model 2 used the same variables except for post-ablation AF burden. By conducting a likelihood ratio test to compare these two models, we find that adding post burden in addition to recurrence and time to recurrence improved the model significantly (Log likelihood −284.38 vs. −289.49, *P*-value = 0.0014; *Table 3*).

**Table 3:** Multivariable models predicting post-ablation AFSS score

	Estimate	SE	*P*-value
Intercept	3.236	1.843	0.082
AF recurrence	−0.146	1.300	0.911
Time to AF recurrence	0.0001	0.004	0.975
Smartphone AF burden post-ablation	14.168	4.409	0.002
**AIC** = **578.77**			

	Estimate	SE	*P*-value
Intercept	6.332	1.649	0.000
AF recurrence	0.501	1.349	0.711
Time to AF recurrence	−0.008	0.003	0.022
**AIC = 586.97**			

AF, atrial fibrillation; AFSS, atrial fibrillation severity scale; AIC, akaike information criterion; SE, standard error.

### Antiarrhythmic drugs post-ablation

The most common drugs used in our patient population were flecainide (12.2%), digoxin (11.2%), and amiodarone (10.2%; *Table 4*). In the DECAAF II trial protocol, the use of antiarrhythmic drugs (AADs) post-ablation was left to the discretion of the treating physician, including the decision to start the drug, type of drug, and timing of drug initiation post-ablation. Importantly, we found that the use of AADs was not different between the two groups (*Table 4*). In addition, the multivariable model did not show any significant impact of AAD use on the drop in AF burden.

**Table 4:** Antiarrhythmic drug use post-ablation in both the recurrence and non-recurrence groups

	No recurrence group (*n* = 29) *n* (%)	Recurrence group (*n* = 69) *n* (%)	Total (*n* = 98) *n* (%)	*P*-value
Any antiarrhythmic drug	12 (41.4)	30 (43.5)	42 (42.9)	0.85
Amiodarone	3 (10.3)	7 (10.1)	10 (10.2)	0.98
Digoxin	5 (17.2)	6 (8.7)	11 (11.2)	0.22
Dofetilide	0 (0.0)	4 (5.8)	4 (4.1)	0.19
Dronedarone	0 (0.0)	1 (1.4)	1 (1.0)	0.51
Flecainide	3 (10.3)	9 (13.0)	12 (12.2)	0.71
Propafenone	0 (0.0)	1 (1.4)	1 (1.0)	0.51
Sotalol	3 (10.3)	6 (8.7)	9 (9.2)	0.80

### Atrial fibrillation burden post-ablation and time to recurrence

We performed Cox analysis, which showed that higher AF burden post-ablation is strongly associated with earlier AF recurrence (*β* coefficient = 6.099, *P* < 0.001).

## Discussion

Our analysis has revealed several significant findings. First, our study shows that catheter ablation for AF reduces AF burden, irrespective of conventional binary recurrence status. Secondly, we found that a higher degree of atrial fibrosis is associated with a lower degree of improvement in AF burden following ablation. Thirdly, we observed a reduction in symptom severity following ablation. Lastly, there is a correlation between the decrease in symptom severity and the decrease in AF burden following the ablation procedure.

In electrophysiology trials, the occurrence of AF after an ablation procedure is typically categorized as a binary outcome. This involves patients either experiencing AF recurrence or not, with the method used to detect recurrence determined by techniques, such as 24 h Holter monitoring, symptom recording, or transtelephonic transmission of ECG recordings. Despite their established effectiveness, these methods only offer a static view of the constantly changing nature of cardiac arrhythmias. Recently, there has been a paradigm shift in defining AF as a disease reflected by its burden, such as the percentage of time spent in AF^6–10^ or symptom burden.^11^ Various tools can be used to assess AF burden, including continuous monitoring by subcutaneous insertable cardiac monitors, which provide the most comprehensive picture of AF but require invasive implantation and carry a small risk of infection.^12^ Our study offers a non-invasive, widely available, and cost-effective alternative using single-lead ECG technology, which has high sensitivity and specificity in detecting AF. Intermittent 19 min recordings using single-lead ECG showed comparable detection capacity to 24 h Holter monitoring.^13^ Additionally, smartphone ECG has demonstrated significant benefits in detecting AF compared with usual monitoring methods, doubling treatment-relevant AF detection.^14^ It is widely accepted across multiple studies that a higher AF burden is an independent predictor of ischaemic stroke and cardiovascular mortality,^15^ highlighting the clinical significance of assessing AF burden. Furthermore, the temporal and diurnal variations in AF patterns have shown significant clinical correlates. Results from the RACE V trial demonstrated that patients with a predominant pattern of AF occurrence (daytime or nocturnal) have a better comorbidity profile compared with those with a mixed pattern of AF.^16^

Catheter ablation has been demonstrated to be an effective therapeutic intervention for AF. It has been demonstrated to restore sinus rhythm, reduce symptom burden, and enhance quality of life.^2^ Andrade *et al*.^17^ found that early ablation reduced the progression from paroxysmal AF to persistent AF, characterizing it as a disease-modifying procedure. Our results suggest that catheter ablation is indeed a disease-modifying procedure since it substantially decreases AF burden after the procedure, despite the recurrence of arrhythmia. This could be explained by the fact that ablation targets and eliminates AF triggers during PVI and modifies the substrate surrounding the pulmonary veins.^18^ Additionally, it could be attributed to the fact that ablation creates a modulation of the autonomic nervous system through vagal denervation.^19^ Multiple studies have reported the positive impact of ablation on structural and functional remodelling.^20^ We also report that ablation decreases symptom burden in patients with persistent AF, which is consistent with prior research.^11^ Furthermore, we demonstrate that symptom improvement after the procedure is highly correlated with the reduction of AF burden.

The predictors of successful ablation have been extensively investigated in the existing literature. Voskoboinik *et al*.^21^ conducted a meta-analysis and identified age and arrhythmia recurrence during the blanking period as significant predictors of arrhythmia recurrence. Gender has also been shown to be a significant predictor of ablation success.^22^ However, we did not observe this trend in our study since our sample was predominantly male (77.3%). Furthermore, our study population was relatively young, with a mean age of 62 years. Creta *et al*.^23^ found that diabetes is a significant predictor of ablation outcomes, with diabetic patients being more likely to experience procedure failure. Additionally, LA fibrosis, as detected by late gadolinium enhancement MRI, has been found to be a strong predictor of ablation failure in the DECAAF I trial.^24^ Using a multivariable model, we demonstrate that Utah Stage 4 (fibrosis > 35%) is an independent predictor of a minimal decrease in AF burden following ablation, which is in line with prior research. This finding raises the question of whether patients with a high degree of fibrosis should undergo ablation.

## Conclusions

To summarize, our study’s findings suggest that catheter ablation is effective in reducing AF burden despite arrhythmia recurrence after the procedure. We also observed a strong correlation between the reduction in AF burden and the improvement in symptom burden following the procedure. Additionally, our study highlights that elevated LA fibrosis significantly impedes the decrease in AF burden following catheter ablation.

### Limitation

This study is a retrospective *post hoc* analysis of the DECAAF II trial, which introduces inherent bias by design. Additionally, the design of the DECAAF II trial did not include a control group that abstained from ablation, limiting our ability to compare results. While the ECG monitoring was comprehensive, with patients submitting daily ECG strips through a handheld device, this monitoring method is less rigorous when compared with implantable loop recorders. In addition, we were unable to perform a one-to-one comparison with an implanted loop recorder (ILR) or Holter monitor. Prior research evaluating AF recurrence rates following ablation with implantable loop recorders has demonstrated higher recurrence rates than previously reported with intermittent monitoring. Furthermore, ECG reporting compliance decreased over the trial duration, implying that the actual recurrence rate of AF and AF burden could have been underestimated in the current study. Additionally, the study population was predominantly male (72.6%) and relatively young, which may have influenced the results when compared with previous studies. Moreover, patients in the no-recurrence group submitted significantly fewer ECG strips for analysis than those in the recurrence group, which may have biased the primary outcome of the study, given that a more complete monitoring could detect more arrhythmia. This could be attributed to the possibility that some patients in the recurrence group experienced symptomatic arrhythmia, which may have resulted in a higher ECG submission rate.

## Data Availability

The data will be made available upon reasonable request.
